# Nitric Oxide (NO) Scaffolds the Peroxisomal Protein–Protein Interaction Network in Higher Plants

**DOI:** 10.3390/ijms22052444

**Published:** 2021-02-28

**Authors:** Francisco J. Corpas, Salvador González-Gordo, José M. Palma

**Affiliations:** Antioxidant, Free Radical and Nitric Oxide in Biotechnology, Food and Agriculture Group, Department of Biochemistry, Cell and Molecular Biology of Plants, Estación Experimental del Zaidín, Spanish National Research Council (CSIC), C/ Profesor Albareda, 1, E-18008 Granada, Spain; salvador.gonzalez@eez.csic.es (S.G.-G.); josemanuel.palma@eez.csic.es (J.M.P.)

**Keywords:** antioxidant, catalase, nitric oxide, peroxisome, reactive nitrogen species, *S*-nitrosation, tyrosine nitration

## Abstract

The peroxisome is a single-membrane subcellular compartment present in almost all eukaryotic cells from simple protists and fungi to complex organisms such as higher plants and animals. Historically, the name of the peroxisome came from a subcellular structure that contained high levels of hydrogen peroxide (H_2_O_2_) and the antioxidant enzyme catalase, which indicated that this organelle had basically an oxidative metabolism. During the last 20 years, it has been shown that plant peroxisomes also contain nitric oxide (NO), a radical molecule than leads to a family of derived molecules designated as reactive nitrogen species (RNS). These reactive species can mediate post-translational modifications (PTMs) of proteins, such as *S*-nitrosation and tyrosine nitration, thus affecting their function. This review aims to provide a comprehensive overview of how NO could affect peroxisomal metabolism and its internal protein-protein interactions (PPIs). Remarkably, many of the identified NO-target proteins in plant peroxisomes are involved in the metabolism of reactive oxygen species (ROS), either in its generation or its scavenging. Therefore, it is proposed that NO is a molecule with signaling properties with the capacity to modulate the peroxisomal protein-protein network and consequently the peroxisomal functions, especially under adverse environmental conditions.

## 1. Overview of the Diversity of Peroxisomes in Eukaryotic Cells

**Figure 1:**
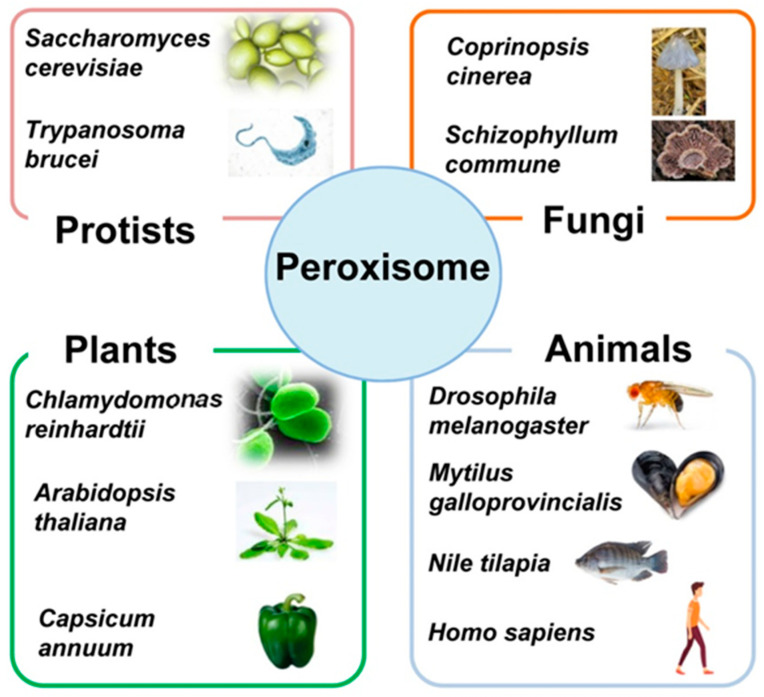
Peroxisomes are single-membrane subcellular organelles that appear in almost all eukaryotic cells from the four kingdoms: protists, fungi, plants and animals.

Peroxisomes are single-membrane subcellular organelles that appear in almost all eukaryotic cells of the four kingdoms including protists, fungi, plants and animals [1,2,3,4,5,6]. The first structural identification of these organelles was done by electron microscope analyses in mouse kidney cells [7]. Years later, those new structures were isolated and biochemically characterized in rat liver being recognized as a new cellular compartment having a prominent hydrogen peroxide (H_2_O_2_) metabolism [8,9]. From that time, studies on peroxisomes were gradually extended to other organisms [1,10,11,12,13,14,15,16,17,18]. Figure 1 shows some representative species, belonging to each kingdom, where peroxisomes have been studied, and Table 1 displays various examples of the diversity of functions exerted by peroxisomes depending on the organism.

**Figure 2:**
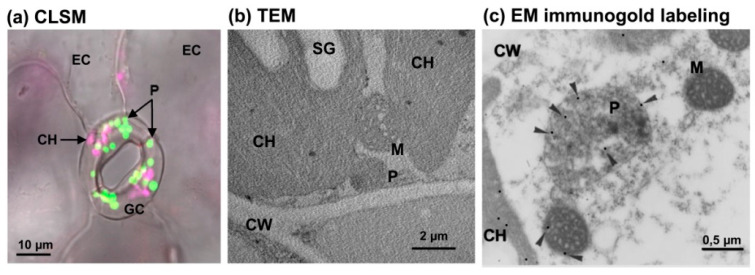
Images of leaf peroxisomes. (**a**) Confocal laser scanning microscope (CLSM) picture of the in vivo detection of peroxisomes (bright green) and chloroplasts (bright purple) in guard cells from *Arabidopsis thaliana* seedlings expressing the cyan fluorescent protein (CFP) fused to a peroxisome targeting signal 1 (PTS1). (**b**) Representative transmission electron microscope (TEM) micrograph of a thin section of a leaf from the halophyte *Cakile maritima* L. where the different subcellular compartments are observed (unpublished results). (**c**) Electron microscope (EM) immunocytochemical localization of NADP-isocitrate dehydrogenase (NADP-ICDH) in pea (*Pisum sativum* L.) leaves. Arrowheads indicate 15-nm gold particles. Reproduced with permission from [25]. Copyright American Society of Plant Biologists. CH, Chloroplast. CW, cell wall. EC, epidermal cells. GC, guard cells. M, mitochondrion. P, peroxisome. SG, starch granule.

At the morphological level, such as it has been mentioned, peroxisomes seem to have a very basic structure because they have a single lipid bilayer membrane that engulfs an amorphous matrix which sometimes has protein crystals. Figure 2 shows the visualization of leaf peroxisomes from different plant species including transgenic *Arabidopsis thaliana* expressing cyan fluorescent protein (CFP) through the addition of peroxisomal targeting signal 1 (PTS1), thus using a confocal scanning laser microscope (CLSM) peroxisomes appear as fluorescence punctuates (Figure 2a); in *Cakile maritima* using a transmission electron microscope (TEM) where the peroxisome is close to chloroplast and mitochondrion (Figure 2b); and in pea (*Pisum sativum* L.) leaf peroxisome housing the NADP-isocitrate dehydrogenase (NADP-ICDH) detected by its immunolocalization by TEM (Figure 2c). Nevertheless, research on peroxisome morphology and biogenesis is a very active research area [39,40,41,42]. Thus, a recent report, using Arabidopsis seedlings, suggests the existence of internal membranes in peroxisomes which open new questions about the morphological complexity which involves protein compartmentation and lipid mobilization [43].

At the biochemical level, it is generally accepted that all peroxisomes have as essential enzymatic constituents the antioxidant catalase and H_2_O_2_-producing flavin oxidases [44,45,46]. However, the enzymatic components can be different depending on the organism, organs, stage of development and environmental cellular conditions. This indicates the metabolic plasticity of peroxisomes and reflects their functional transformation and complexity. Even though, new unexpected enzymes are still located in peroxisomes. For example, the chorismate synthase which catalyzes the last step of the conversion of 5-enolpyruvylshikimate 3-phosphate to chorismate (an essential precursor of Tyr and Phe biosynthesis) in the shikimate pathway, has been very recently found to be also in peroxisomes from petunia (*Petunia hybrida*) plants [30]. Recently, it has been also found that pseudouridine, which is a nucleoside modification that occurs in both noncoding RNAs and mRNAs, is catabolized in Arabidopsis peroxisomes by a pseudouridine kinase (PUKI) [47].

Although the question “What is a peroxisome?” is no new [48], these organelles have received a diverse names from their discovery such as microbody, glycosome, glyoxysomes, Woronin bodies, unspecialized peroxisomes, root and leaf peroxisomes, and gerentosomes, among others [10,12,49,50,51]. However, at present, the recommended canonical name in any eukaryotic organism is peroxisome [2,52].

## 2. Hydrogen Peroxide (H_2_O_2_) and Nitric Oxide (NO) Metabolism in Plant Peroxisomes

In-plant peroxisomes, the metabolism of H_2_O_2_ and other ROS is very active being glycolate oxidase or acyl-CoA oxidase two enzymes involved in photorespiration and β-oxidation, respectively, some of the most representative H_2_O_2_-generating enzymes. However, there are other pathways which involve the generation of H_2_O_2_ such as purine and polyamine metabolism as well as other enzymes including sulfite oxidase or sarcosine oxidase (for a more detailed review see [53]). Besides catalase as the main antioxidant enzyme, plant peroxisomes harbor a significant battery of enzymatic and non-enzymatic antioxidant systems present in both matrix and membrane-bound to keep under control the ROS production under physiological and stressful conditions. Among these enzymatic antioxidants, it could be mentioned the superoxide dismutase (SOD) [54,55,56] and components of the ascorbate-glutathione cycle including ascorbate peroxidase (APX) [56,57,58,59,60], monodehydroascorbate reductase (MDAR) [61,62], dehydroascorbate reductase (DHAR) [63,64,65] and glutathione reductase (GR) [53]. Additionally, the non-enzymatic antioxidant ascorbate and glutathione (GSH) have been also reported to be part of the peroxisomal metabolite profile [66,67,68]. The relevance of the peroxisomal ROS metabolism seems to be most prominent under adverse circumstances when plants undergo either abiotic stressful conditions [69,70] or during their interactions with pathogen microorganisms [26,27,64].

Such as it has been previously mentioned, catalase is the most representative and abundant antioxidant enzyme of peroxisomes, and one of the most studied enzymes so far by the scientific community. Plant catalases use the archetypical peroxin 5 (PEX5) receptor for importing events within the organelle, just like most peroxisomal proteins harboring a PTS1 do [71]. Interestingly, in higher plants, catalase also possesses a degenerated PTS1 and its import depends on the neighboring C-terminal amino acid residues [72,73,74,75]. In this sense, it should be highlighted that in animal cells under stress conditions, an inefficient import mechanism involving the PEX5 receptor provokes that catalase remains in the cytosol where it exerts its antioxidant function. This constitutes an additional molecular mechanism of cellular protection against oxidative stress [76].

Nitric oxide is catalogued as a radical molecule because it has an unpaired electron in its π orbital. This chemical characteristic of NO has conferred diverse properties which makes NO a molecule with signal functions. It has a family of derived molecules designated as RNS such as peroxynitrite (ONOO^−^), dinitrogen trioxide (N_2_O_3_), nitrogen dioxide (NO_2_), *S*-nitrosoglutathione (GSNO) or *S*-nitrosothiols (SNOs), among others. RNS can interact with numerous macromolecules including proteins, fatty acids or nucleic acids affecting their biological functions [77]. In the case of proteins, NO regulates their functions through posttranslational modifications (PTMs), mainly tyrosine nitration, *S*-nitrosation and metal nitrosylation. Tyrosine nitration involves the addition of a nitro (-NO_2_) group of tyrosine residues being an irreversible process and, in general, it has associated a loss of function of the affected proteins [78,79]. *S*-nitrosation comprises the covalent attachment of an NO group to the thiol (-SH) side chain of cysteine (Cys) residues. This interaction is a reversible process being a mechanism of redox regulation of the target proteins. So far protein *S*-nitration has shown to affect either negatively or positively the function of the target protein. On the other hand, protein metal nitrosylation comprises the interaction between NO with transition metals (Fe/Cu) present in metalloproteins such as cytochrome *c* oxidase or catalase.

Unlike animal cells in which NO is generated by the different isoforms of the enzyme nitric oxide synthase (NOS), in plants, there is still a great controversy regarding the enzymatic source responsible for NO generation. Currently, there are two main enzymatic candidates, nitrate reductase (NR) and a still unknown system whose activity is similar to the NOS in animal cells since the generation of NO depends on L-arginine using NADPH as a source of reducing power [80,81]. In-plant peroxisomes, there is not any report about the presence of NR, however, there is accumulative experimental evidence of the presence of an L-arginine dependent NO synthase activity which has all the biochemical requirements (NADPH, calmodulin, calcium, FAD, FMN, BH_4_) of the animal NOS [82,83,84], although its identity has not been elucidated yet. Previously, it was hypothesized that an alternative NO source in the peroxisome was the enzyme xanthine oxidoreductase (XOD) since its presence in plant peroxisomes was early demonstrated [85]. However, there is no experimental evidence that this XOD is responsible for the generation of NO in peroxisomes from plant origin. On the other hand, the generation of NO from other non-enzymatic processes could not be ruled out, for example, polyamines and polyamides. However, to our knowledge, there is no experimental evidence that in plant peroxisomes they are these sources of NO either. Independently of the identity of the NO source, the generation of this radical molecule into plant peroxisomes have been demonstrated by different biochemical and cellular approaches such as electron paramagnetic resonance spectroscopy (EPR) as well as fluorometric analyses [82]. Complementarily, it has been demonstrated the presence of peroxynitrite in these organelles [86] as well as the identification of peroxisomal proteins which undergo NO-derived PTMs, either *S*-nitrosation or Tyr-nitration [77]. Table 2 summarizes all plant peroxisomal proteins identified so far that undergo NO-mediated PTMs and how they affect their function. These PTMs are irrefutable pieces of evidence of the presence and relevance of NO in peroxisomal metabolism.

## 3. NO and Protein–Protein Interactions (PPIs) in Plant Peroxisomes

**Figure 3:**
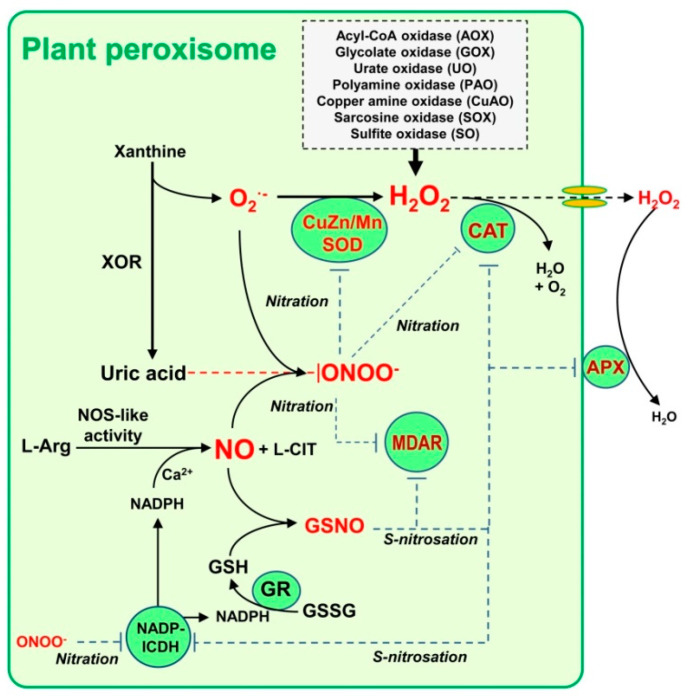
Working model showing the existing interactions between NO metabolism and reactive oxygen species in leaf peroxisomes based on available experimental data. Plant peroxisomes contain a team of H_2_O_2_-generating enzymes involved in diverse pathways. Xanthine oxidoreductase (XOR) activity catalyzes the conversion of xanthine to uric acid with the concomitant generation of superoxide radical (O_2_^•−^) which immediately dismutates to H_2_O_2_ by the action of CuZn/Mn-superoxide dismutase (CuZn/Mn-SOD). Then, this H_2_O_2_ is removed by catalase (CAT) activity. Additionally, a fraction of this H_2_O_2_ can be decomposed by the membrane-bound ascorbate peroxidase (APX). An L-arginine (L-Arg) and Ca^2+^ dependent NOS-like activity generates L-citrulline (L-CIT) plus NO. This NO interacts chemically with O_2_^•−^ to generate peroxynitrite (ONOO^−^), a strong nitrating compound that facilitates posttranslational modifications (PTMs) such as tyrosine nitration. But ONOO^−^ can be simultaneously scavenged by the uric acid formed through the XOR activity (dashed red line). NO may react with reduced glutathione (GSH) to produce *S*-nitrosoglutathione (GSNO), a NO donor which facilitates *S*-nitrosation. GSH is restored by glutathione reductase (GR) which needs NADPH provided by several NADPH-generating enzymes (NADPH-ICDH, G6PDH and 6PGDH). Uric acid is an ONOO^−^ scavenger which allows controlling the effect of NO metabolism. Among the peroxisomal targets of NO-derived PTMs identified so far (see Table 2) it is remarkable that CAT, CuZn-SOD and monodehydroascorbate reductase (MDAR) can undergo an inhibitory effect either by nitration or *S*-nitrosation. The dashed blue line denotes an inhibitory effect. Modified from [53].

Based on the available information (Table 2), Figure 3 shows a working model of the metabolism of peroxisomal NO and how it can modulate the ROS metabolism through NO-derived PTMs. Thus, an L-arginine, NADPH and Ca^2+^ dependent NOS-like activity generates L-citrulline plus NO which can immediately react with superoxide anion (O_2_^•−^), generated by the enzyme XOD, at a rate constant (k) of 1.9 10^10^ M^−1^ s^−1^ to produce peroxynitrite (ONOO^−^) [87], or it could be dismutated to H_2_O_2_ by either a CuZn- or a Mn-superoxide dismutase (CuZn-SOD/Mn-SOD, respectively) isozymes. Peroxynitrite is a nitrating molecule that facilitates protein Tyr-nitration [77,88]. NO can also interact with reduced glutathione (GSH) to form GSNO, a NO donor which mediates processes of protein *S*-nitrosation and transnitrosation [89,90]. GSH is regenerated by the peroxisomal glutathione reductase (GR) which requires NADPH as an electron donor, a cofactor supplied by several peroxisomal NADPH-generating enzymes such as NADP-isocitrate dehydrogenase (ICDH) and both dehydrogenases of the oxidative step of pentose phosphate pathway, glucose-6-phosphate dehydrogenase (G6PDH) and 6-phosphate dehydrogenase (6PGDH) [25,91,92]. In the case of NADP-ICDH, it has been shown that its activity is regulated by NO [93]. On the other hand, XOD, as part of the purine metabolism, generates uric acid which is an efficient ONOO^−^ scavenger [94,95]. Both uric acid and ONOO^−^ have been detected in leaf peroxisomes [86,96]. The relevance of uric acid has been shown by genetic approaches because its impairment affects negatively the Arabidopsis peroxisome function and seedling establishment [97]. On the other hand, under cadmium-induced oxidative stress, where many protein antioxidants are affected, there is an increase of peroxisomal ONOO^−^ content [86]. Conversely, plant peroxisomes contain a significant number of H_2_O_2_-generating enzymes involved in diverse pathways including photorespiration, β-oxidation, purine and polyamine metabolism among others. The H_2_O_2_ pool content is mainly kept under control by catalase (CAT) but if there is an overproduction due to adverse cellular circumstances this H_2_O_2_ could be release out of the organelle and its level is controlled by the membrane-bound ascorbate peroxidase (APX). These examples support the significance of these reactive species in the plant peroxisomal metabolism. Besides, all these intricate interactions suggest that there is a mechanism of peroxisomal auto-regulation between ROS and RNS metabolism where NO functions upstream of ROS.

**Figure 4:**
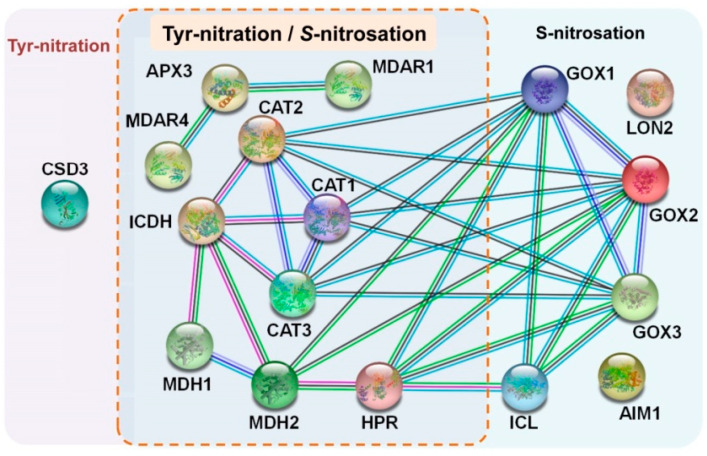
Predicted computational protein-protein interactions (PPIs) network of peroxisomal proteins undergoing NO-mediated post-translational modifications (either tyrosine nitration or S-nitrosation) in Arabidopsis. The color code for depicted lines is as follows: pink, experimental evidence; blue, database evidence; green, neighboring genes; dark-blue, gene co-occurrence; black, displays co-expression; purple, protein homology. The analysis was performed using STRING v11.0 with minimum required interaction score set in “medium confidence” (0.400). CAT, catalase. CSD3, CuZn-SOD. GOX, glycolate oxidase. HPR, hydroypyruvate reductase. ICDH, NADP-isocitrate dehydrogenase. ICL, isocitrate lyase. LON2, Lon protease homolog 2. MDAR, monodehydroascorbate reductase. MDH, malate dehydrogenase.

Considering the pool of peroxisomal proteins, target of NO-derived PTMs (nitration and *S*-nitrosation) and to better understanding the dynamic metabolic processes among them, it was analyzed the protein-protein interaction (PPI) network of these proteins which are regulated by NO. Figure 4 displays the analysis of the predicted PPI network using the STRING database, version 11.0 (https://string-db.org/ accessed on 28 February 2021) [98] which allows to visualize/evaluate the functional association of some peroxisomal proteins which undergo PTMs promoted by RNS. This allows highlighting that among the identified peroxisomal proteins the majority of them are targets of both NO-mediated PTMs (Tyr-nitration and *S*-nitrosation), being most of them antioxidant enzymes such as catalase, APX and MDAR. This seems to suggest that NO could be an upstream signal of peroxisomal ROS metabolism.

## 4. Can NO Be a Signal Which Scaffolds Peroxisomal Function?

Based on the available information about the relevance of NO to regulate the plant peroxisomal antioxidant system and consequently the level of ROS, it could be proposed that NO is a signal molecule which scaffolds the peroxisomal functions. Thus, there are several pieces of evidence which could support this idea. Such as it has been mentioned before, CAT is a key peroxisomal antioxidant enzyme which activity is highly regulated by diverse PTMs mediated by RNS [99] and, consequently, influences the endogenous H_2_O_2_ content. On the other hand, it has been identified that the Arabidopsis CAT3 can mediate a process of trans-nitrosation of the enzyme GSNO reductase (thus being also designated as ROG1, Repressor of GSNOR1) and consequently it mediates NO signaling [100,101]. In fact, CAT3/ROG1 was shown to be localized to the peroxisome, cytoplasm, and plasma membrane [102,103] and in Arabidopsis plants under hypoxia conditions, this NO-mediated PTM triggered the specific degradation of GSNOR via autophagy [104]. Moreover, a recent report suggests that CAT has a transnitrosation activity that regulates the stability of GSNOR1 by its modification [101].

Pexophagy is a process that allows removing damaged peroxisomes and different reports have shown that ROS metabolism is directly involved because oxidized and, consequently, damaged peroxisomal proteins are eliminated via autophagy [105,106,107,108,109,110]. This is in good agreement with previous reports which indicate both malate synthase and catalase are susceptible to be oxidized by H_2_O_2_ and prone to be further degraded [111]. Although catalase activity is considered a very stable enzyme over broad temperature and pH range, there is also some data indicating that catalase could undergo an oxidation process [112,113]. For example, in this latter case, an in vitro studies using pepper fruit extracts incubated with increased H_2_O_2_ concentrations (1–50 mM) showed that treatments higher than 5 mM H_2_O_2_ for 30 min at 25ºC provoked an activity inhibition up to 40%. Furthermore, analysis of these samples through non-denaturing PAGE and further in-gel catalase activity staining showed that in samples incubated with the highest H_2_O_2_ concentrations, the catalase isozymes had higher electrophoretic mobility, and this was consistent with the oxidation of the protein [113]. It has been also found that in peroxisomes from Arabidopsis autophagy-related mutants designated as ATG, there is an accumulation of inactive CAT and in the case of the *atg2* mutant, the damaged peroxisomes are removed by pexophagy [106,114]. Likewise, other *atg* mutants (*atg5*, *atg7*, *atg10*, and *atg12*) exhibit increased aggregation of peroxisomes in guard cells. These mutants display higher ROS content which compromised stomatal opening, indicating that autophagy affects guard cell ROS homeostasis and as a result stomata opening [115].

Recently, it has been established a close interaction between CAT and nitrate reductase (NR) in cassava (*Manihot esculenta*) plants as a mechanism of defence against the bacteria *Xanthomonas axonopodis* since these enzymes are involved in the mechanism of regulation of the H_2_O_2_ and NO content, respectively. Accordingly, it has been found that the transcription factor RAV5 acts at two levels: 1) RV5 activates the NR transcript and consequently the NO content; and 2) RV5 interacts with CAT1 triggering its activity inhibition which, in turn, elevates the H_2_O_2_ contents [116]. Therefore, this mechanism to increase of NO and H_2_O_2_ content is an excellent example of coordination of the cellular mechanism of defence where peroxisomes play a critical function.

## 5. Conclusions and Future Trials

**Figure 5:**
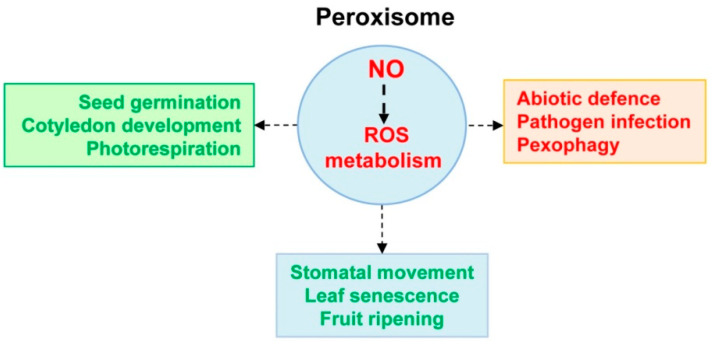
Working model for the implication of the peroxisomal NO/ROS metabolism in physiological and stressful plant processes. Based on the available information, it is suggested that NO throughout its derived PTMs (*S*-nitrosation and tyrosine nitration) can modulate ROS metabolism. Thus, some antioxidant enzymes can undergo either *S*-nitrosation or nitration affecting their activity and, consequently, modulating the content of molecules such as NO, O_2_^•–^ or H_2_O_2_. Therefore, the relevance of these NO-derived PTMs could be different according to the distinct physiological or stressful processes where the levels of NO and ROS could be significantly affected.

Peroxisomes play multiple functions in higher plants as a consequence of their metabolic plasticity which depends on the plant species, organs, stage of development and environmental conditions. Besides the “classical” enzymatic components of peroxisomes during the last years, the identification of new enzymatic and non-enzymatic components indicates that this organelle is more complex than was expected considering its simple morphological structures. However, it should be pointed out that unlike chloroplasts and mitochondria that contain their own DNA, peroxisomes do not harbor genetic material, so all their proteins are encoded in the nucleus and have to be imported into the organelle once they have been synthesized in the cytosol. Nowadays, it is known that plant peroxisomes have an active nitro-oxidative metabolism where the *modus operandi* of NO seems to act as an upstream signal because it can modulate the activity of significant peroxisomal components, specifically those related to ROS metabolism. Consequently, it could be proposed that NO thought PTMs, *S*-nitrosation and/or nitration, can be a key element to integrate a complex peroxisomal protein-protein network that could have either synergistic or antagonistic interactions which other molecules such as H_2_O_2_. All these molecular connections provide exciting avenues for future research in plant peroxisomes which subsequently affect whole-cell homeostasis and all functions where peroxisomes are involved. Figure 5 displays a simple model which summarizes the peroxisomal NO/ROS metabolism and its implication in physiological and stressful plant processes.

## Figures and Tables

**Table 1:** Diversity of functions exerted by peroxisomes depending on the organism.

Organism/Organ (Species)	Peroxisomal Function	Ref.
Free-living marine diplonemid (*Diplonema papillatum*)	Peroxisome undergoes remodeling metabolism involving the housing gluconeogenesis	[19]
Ascomycete (*Sclerotinia sclerotiorum*)	Peroxisome is involved in the fungi sexual development	[20]
Fungus (*Alternaria alternate*)	Peroxisome of the fungus is necessary for its pathogenesis in citrus	[21]
*Chlamydomonas*	Peroxisomal malate dehydrogenase 2 connects lipid catabolism to photosynthesis	[22,23]
Pea leaves (*Pisum sativum*)	Peroxisomes involve in leaf senescence	[24,25]
Leaf tomato (*Solanum lycopersicum*)	Peroxisomes involve in pathogen defence	[26,27]
Leaves (*Arabidopsis thaliana*)	Peroxisomal NADP-isocitrate dehydrogenase is required for stomatal movementPeroxisomal trehalose-6-phosphate phosphatase I is essential for flowering and development	[28,29]
Petunia (*Petunia hybrida*)	Peroxisomal and chloroplastic chorismate synthase, involved in shikimate pathway, are need for flower development	[30]
Mussels (*Mytilus edulis*)	Peroxisome proliferation in response to environmental pollutants	[31]
Nile tilapia (*Oreochromis niloticus*)	Impaired of peroxisomal fat oxidation induces hepatic lipid accumulation and oxidative damage	[32]
Rat liver (*Rattus norvegicus*)	Peroxisomes participate in the metabolism of xenobiotic acyl compounds	[33]
Human (*Homo sapiens*)	Defects in genes encoding peroxisomal proteins lead to a variety of human diseases. For example, X-linked adrenoleukodystrophy, acatalasemia, cerebro-hepato-renal syndrome, etc.Host defense	[34,35,36,37,38]

**Table 2:** Identified peroxisomal enzymes target of diverse NO-derived posttranslational modifications (tyrosine nitration or *S*-nitrosation) and the effect on their function.

Peroxisomal Enzyme	NO-Derived PTM	Effect on Activity
**Antioxidants**		
Catalase (CAT)	Tyr-nitration S-nitrosation	Inhibition Inhibition
Monodehydroascorbate reductase (MDAR)	Tyr-nitration S-nitrosation	Inhibition Inhibition
Ascorbate peroxidase (APX)	Tyr-nitration *S*-nitrosation	Inhibition Activation
CuZn-superoxide dismutase (CSD3)	Tyr-nitration	Inhibition
**Photorespiration**		
Hydroxypyruvate reductase (HPR)	Tyr-nitration *S*-nitrosation	Inhibition Inhibition
Glycolate oxidase (GOX)	*S*-nitrosation	Inhibition
**Fatty acid β-oxidation**		
Malate dehydrogenase (MDH)	Tyr-nitration S-nitrosation	Inhibition Inhibition
Isocitrate lyase (ICL)	*S*-nitrosation	Not reported
Multifunctional protein AIM1 isoform	*S*-nitrosation	Not reported
**Glyoxylate cycle**		
Isocitrate lyase (ICL)	*S*-nitrosation	Not reported
**Peroxisomal protein import**		
Lon protease homolog 2	*S*-nitrosation	Not reported
**NADPH supply**		
NADP-isocitrate dehydrogenase (NADP-ICDH)	Tyr-nitration *S*-nitrosation	Inhibition Inhibition
